# Supplementary material for: Mechanisms governing the pioneering and redistribution capabilities of the non-classical pioneer PU.1
Source: Nat Commun. 2020 Jan 21;11:402. doi: 10.1038/s41467-019-13960-2 (PMC6972792; doi:10.1038/s41467-019-13960-2)
Supplement: Supplementary file 7 — Source data [file 41467_2019_13960_MOESM7_ESM.zip › Source_Data/FigureS2/FigureS2B_MotifScanOutput/KmeansCluster13/homerResults/motif2.similar.html]

motif2

## Information for motif2

T
A
C
G
T
C
A
G
C
G
T
A
C
T
G
A
T
C
G
A
A
G
T
C
A
G
T
C
C
G
T
A
G
T
A
C
G
T
C
A
  
Reverse Opposite:  

C
A
G
T
A
C
T
G
G
C
A
T
T
C
A
G
A
C
T
G
A
G
C
T
G
A
C
T
G
C
A
T
A
G
T
C
A
T
G
C
  

|  |  |
| --- | --- |
| p-value: | 1e-127 |
| log p-value: | -2.935e+02 |
| Information Content per bp: | 1.656 |
| Number of Target Sequences with motif | 548.0 |
| Percentage of Target Sequences with motif | 32.39% |
| Number of Background Sequences with motif | 4629.6 |
| Percentage of Background Sequences with motif | 10.27% |
| Average Position of motif in Targets | 101.3 +/- 53.1bp |
| Average Position of motif in Background | 100.7 +/- 62.8bp |
| Strand Bias (log2 ratio + to - strand density) | 0.1 |
| Multiplicity (# of sites on avg that occur together) | 1.19 |
| Motif File: | file (matrix) reverse opposite |

### Similar de novo motifs found

|  |  |  |  |  |  |  |  |
| --- | --- | --- | --- | --- | --- | --- | --- |
| Rank | Match Score | Redundant Motif | P-value | log P-value | % of Targets | % of Background | Motif file |
| 1 | 0.984 | G C A T C G T A T G C A A G T C A G T C C G T A A G T C G T C A | 1e-125 | -289.605708 | 37.88% | 13.87% | motif file (matrix) |
| 2 | 0.977 | G A C T A C G T C A T G G C A T A T C G A T C G A C G T G C A T C G T A | 1e-110 | -254.645476 | 35.28% | 13.25% | motif file (matrix) |
| 3 | 0.981 | C A T G T A G C G C T A C T G A T C G A A G T C A G T C C T G A A T G C G T C A T G C A | 1e-102 | -235.387813 | 27.78% | 9.07% | motif file (matrix) |
| 4 | 0.969 | A C G T A C T G A G C T A C T G A C T G A C G T C G A T | 1e-87 | -201.949467 | 19.74% | 5.38% | motif file (matrix) |
| 5 | 0.806 | G C A T T C G A G C T A G T A C G T A C C T A G G A T C | 1e-76 | -175.021433 | 60.22% | 37.52% | motif file (matrix) |
| 6 | 0.948 | C T G A T C A G T A C G C G T A C T G A T C G A G A T C A G T C C G T A T A G C G T C A T C G A | 1e-67 | -155.860450 | 12.77% | 2.89% | motif file (matrix) |
| 7 | 0.872 | C T G A T A C G T G C A T C A G T C A G G T C A C T G A T C G A T A G C G A T C G C T A T A G C G C T A A T C G | 1e-65 | -150.868172 | 19.21% | 6.41% | motif file (matrix) |
| 8 | 0.843 | C A T G T A C G T C G A C T A G A T C G G C T A C T G A C T G A A G T C G A T C C T G A A T G C G C T A | 1e-50 | -115.133201 | 6.32% | 0.88% | motif file (matrix) |
| 9 | 0.846 | G C T A A C T G A G T C G A C T G C A T T C G A T G C A T A G C A G T C C G T A A G T C G C T A | 1e-34 | -79.124024 | 7.45% | 1.90% | motif file (matrix) |
| 10 | 0.842 | C G T A A T C G A G T C A T G C C T G A A G T C C G T A | 1e-28 | -64.977640 | 25.95% | 15.23% | motif file (matrix) |
| 11 | 0.816 | C G A T A T C G C G A T C A T G T A C G C A G T C A G T C G T A C T G A C T A G G T A C | 1e-26 | -60.825642 | 5.26% | 1.24% | motif file (matrix) |
| 12 | 0.766 | A T G C A G C T A C G T A C G T A C T G G T C A C G A T A T C G C A G T A C T G A C T G A G C T G A C T | 1e-25 | -59.604967 | 7.09% | 2.19% | motif file (matrix) |
